# A situational analysis of HIV and AIDS in Pakistan

**DOI:** 10.1186/1743-422X-8-191

**Published:** 2011-04-25

**Authors:** Muhammad Ilyas, Sultan Asad, Liaqat Ali, Masaud Shah, Sadaf Badar, Muhammad Tahir Sarwar, Aleena Sumrin

**Affiliations:** 1 Bio-Pharmaceutical Lab, National Centre of Excellence in Molecular Biology, University of the Punjab/Centre for Applied Molecular Biology,Ministry of Science and Technology, Lahore, Pakistan

## Abstract

HIV (Human immunodeficiency virus) transmission has been reduced by protected sex and screening of blood products and other body fluids in the developed countries. It has been reported that Pakistan is at high risk of HIV/AIDS infection but presently the prevalence rate is considerably low. The number of reported cases of HIV/AIDS in Pakistan has been continuously increasing since 1987. By 2010 the total number of registered cases has reached to 6000 and this figure is on the rise with the passage of time. Some serious strategies must be implemented to control this deadly disease.

## Situation in Pakistan

Pakistan's first HIV/AIDS case was detected in 1987 [1,2]. The number has been increased according to the annual report of Pakistan National AIDS Control Program (NACP) [3].

Pakistan, located in South Asia, is the sixth most densely packed country populated by 168.79 million people by the end of 2009 [1]. It has four provinces "Sind, Punjab, Khyber Pakhtoonkhwa and Baluchistan. India, China, Iran and Afghanistan are the neighboring countries of Pakistan. The region has a literacy rate of only 54% [4].

**Figure 1:**
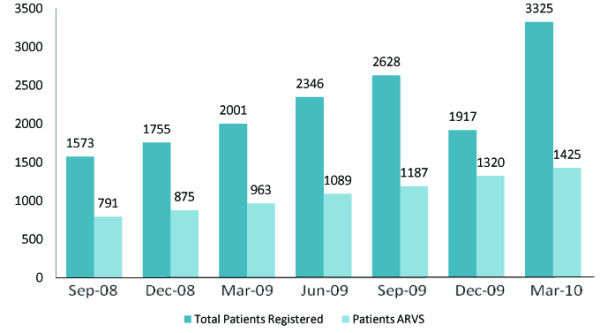
**The number of infected and reported patients infected with HIV**.

Like other Asian countries Pakistan is also HIV epidemic, characterized by different risk factors Formerly Pakistan was considered to be a low prevalence country, but now it is in the group of "Countries in Transition" with a concentrated epidemic among high risk groups, where the AIDS problem is increasing since last five years, according to the private newspaper The News and NACP NIH [5]. The number of infected persons might be running in millions if proper screening is carried out. The behaviors conducive to the spread of HIV infection to young people are curiosity about sex and drugs, negative peer pressure, and economic frustration in Pakistan [6]. Widespread poverty, significant power imbalances in men and women, labor migration, lack of any system to check the HIV positive reported persons, indiscriminate transfusion of unscreened blood, rising number of drug addicts and low condom use rates, are the serious risk factors that put the country in danger of facing a rapid spread of HIV [7]. 9% of the tested injecting drugs users (IDU's) were found to be HIV positive in 2005-2006, this percentage increased to 15.8% in 2006-2007, and it exceeded 20% in 2007-2008 [8]. 97,400 cases of HIV/AIDS were estimated in 2009 and more than 6,000 cases are registered till now (2010). Till March 2010, 3325 patients were registered at national AIDS control center NIH Islamabad. 1425 patients are on ARVS (Figure 1) [9].

The geographic trend of the epidemic has recently expanded from major urban cities and provincial capitals to more rural town and smaller cities. Although national adult HIV prevalence in the general population remains < 0.1%, exceptions were observed as in Jalalpur Jattan (Gujrat) where 90 HIV positive cases were found, out of 342 samples from the general population that included a large number of sex-migrant workers [10]. Among many factors, one important factor attributing to this development is unsafe injecting practices in formal and informal healthcare settings [11]. The mode of HIV/AIDS transmission in Pakistan is largely heterosexual (52.55%), the most commonly reported modes of transmission are contaminated blood or blood products (11.73%) [12]. According to AIDS Asia HIV wide spread is contributed by intravenous drug users (IDU's) (2.02%), male-to-male or homosexual relations (4.55%), mother-to-child transmission (2.2%) and transmission due to undetermined origin (26.9%) along with other factors [13,14]. The major concern in Pakistan is the recent studies which have put the figures in HIV infected IDUs up to 2.5-3.5% during 2004-05. Many of these IDUs are also professional blood donors in a country with inadequate blood transfusion screening; only 50% of the transfused being screened for HIV [15].

**Figure 2:**
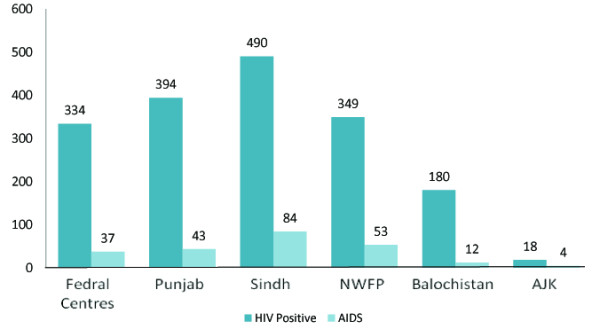
**Epidemiological Situation of HIV/AIDS in Pakistan**. HIV Positive (1765) + AIDS (233) = Total 1998 Cases at Islamabad, Karachi, Lahore, Rawalpindi and FANA (Pakistan Demographic Health Survey, 2007-08).

According to the study conducted by Pakistan Demographic Health Survey, 2007-08, from all the four provinces of Pakistan along with FATA and AJK. In the light of the above said study, total 1998 patients were identified as HIV infected out of which, 1765 were HIV positive only while 233 developed AIDS. The study above said study showed that HIV is most prevalent in the Sindh province while least prevalent in AJK (Figure 2) [16]. 86.8% of reported HIV positive cases are found to be men. Furthermore 51.88% of the HIV infected men fall within the age group of 20-40 years. 24.59% of the reported cases are of unknown origin, 13.20% are females, and 45.10% of the total HIV carriers acquired the disease through sexual contact [17].

Pakistan's National AIDS Control Program (NCP) is one of the pioneer institutions providing free treatment to any person found to be suffering from AIDS through its 20 AIDS Treatment Centers all over the country.

## Future perspectives

1. In a country with low literacy rate one should expect low level of awareness about HIV/AIDS. Awareness needs to be created at all levels especially in rural areas.

2. The government should know its responsibilities and should provide the necessary legal and regulatory frame work for dealing with this silent killer disease.

3. This disease is spreading continuously without knowing the boundaries. Further strategies must be implemented. Otherwise it will be impossible to get rid of this lethal wave.

## Abbreviations

NIH: National Institute of Health; NACP: National AIDS Control Program; ARV: Antiretroviral; FANA: Federally Administered Northern Areas; HIV: Human immunodeficiency virus; AIDS: Acquired Immunodeficiency syndrome.

## Competing interests

The authors declare that they have no competing interests.

## Authors' contributions

MI and AS conceived the study, participated in its design and coordination and gave a critical view of manuscript writing. SA, LA and MS performed and analyzed the data. SB and MTS edited the manuscript and helped MS in literature review. All the authors read and approved the final manuscript.
